# The effect of particle size and water content on XRF measurements of phosphate slurry

**DOI:** 10.1038/s41598-022-21392-0

**Published:** 2022-10-24

**Authors:** Ismail Ben Amar, Mourad Roudjane, Hafid Griguer, Amine Miled, Younès Messaddeq

**Affiliations:** 1 Department of Electrical and Computer Engineering, Université Laval, Quebec City, QC Canada; 2 Innovation Lab for Operations, Mohammed VI Polytechnic University (UM6P), Ben Guerir, Morocco; 3 Center for Optics, Photonics, and Lasers, Université Laval, Quebec City, QC Canada

**Keywords:** Mineralogy, Solid Earth sciences, Chemistry, Analytical chemistry

## Abstract

Phosphate slurries are studied using the XRF technique and the effect of the particle sizes and the water content parameters are analyzed and reported for the first time. Samples of the phosphate slurry with different particle sizes (425 µm, 300 µm, 250 µm, 200 µm, 160 µm and 106 µm) and different water contents (30%, 40%, 50%, 60%) were analyzed using an energy-dispersive X-ray spectrometer (EDXRF). The results show that the relative error of measurement varies with the particle size of the analyzed sample, the water content and the element measured. The relative error increases with the increase of the particle size for the compounds P_2_O_5_, Al_2_O_3_, K_2_O, Cr_2_O_3_, Fe_2_O_3_ and Sr. The ratio between the relative errors related to the maximum and minimum grain sizes was 1.50 for P_2_O_5_, 4.01 for Al_2_O_3_, 15.58 for K_2_O, 1.22 for Cr_2_O_3_, 1.51 for Fe_2_O_3_ and 1.11 for Sr. Alternatively, an opposite evolution has been observed in the case of compounds CaO and SiO_2_. The relative error increases with increasing water content for all compounds existing in the slurry. Depending on the measured compound, the relative error increases by a factor that varies between 1.39 and 2.39. In the case of P_2_O_5,_ the results do not show a clear correlation between the measurement error and the water content. A study will be conducted to investigate the effect of particle size and water content on XRF measurements in the case of phosphate slurry, aiming to develop an online XRF analyzer system for phosphate slurry.

## Introduction

Phosphate mineral slurry is a mixture of dry phosphate and water. It is the raw material to be exploited to produce fertilizers and phosphoric acid. The phosphate slurry is processed to produce phosphorus (P), which is one of the three main nutrients most used in fertilizers (the other two are nitrogen and potassium), and the most important macronutrients essential for the growth and development of a plant. It is a building block of deoxyribonucleic acid (DNA) and ribonucleic acid (RNA) in plant cells and is responsible for energy storage and transfer. Plants acquire all their P from fertilizers in the soil^1^.

The quality of the phosphate slurry is checked regularly throughout the production chain. Samples are taken at variable time intervals between 20 and 60 min depending on the slurry processing steps. Then, the samples are sent to the laboratory to evaluate the parameters such the solid content, the distribution of the particle size and the concentration of the chemical elements P_2_O_5_, CaO, SiO_2_, CO_2_, MgO, Cd, organic C, F, Cl, Al_2_O_3_, Fe_2_O_3_, K_2_O, Na_2_O, SO_3_, U, Zn, As, Cu and Mn. These chemical analyses are carried out in the laboratory using conventional analytical methods. These methods are expensive, time-consuming and generate hazardous waste. An online X-ray fluorescence technique (XRF) analysis system could overcome these drawbacks and allow a rapid chemical analysis of the phosphate slurry. A few studies have demonstrated the potential of the XRF technique to analyze dry phosphate rock^2–5^. However, there is a lack of information on the analysis of phosphate slurry using the XRF technique.

This article will explore some challenges of analyzing phosphate slurry using the XRF technique. These challenges are related to the physical parameters of the slurry, namely, the particle size and water content. These parameters affect the accuracy of XRF measurements and more specifically in the case of slurry. Indeed, analyzing the slurry is a challenging process compared to solid analysis, one of these challenges is the absorption of X-rays by the water.

XRF is a rapid technique for quality control of mineral samples^6^. The time required to analyze a sample can be reduced from hours (in the case of conventional analysis methods) to minutes (in the case of XRF)^7^. Like many other techniques, XRF has some limitations regarding the analysis of light elements^8^. In the case of phosphate slurry, the particle size of the sample and the water content may affect the accuracy of the XRF measurements^9–17^. When analyzing samples with different particle size distributions and light elements or having analytes with long-wave characteristic lines (e.g., Si, Al), peak intensities can be attenuated by 30%^18^. Demir et al. (studied the effect of particle size distribution on XRF measurements of cement samples with different grain sizes. The results show a difference between the maximum intensities of the peaks that can reach 17%, when analyzing samples with different particle sizes^19^. In another study, samples of different grain sizes extracted from the Nile River in Egypt were analyzed with the XRF technique. The results show that the XRF intensities of radiation can increase or decrease when decreasing grain size and depending on the atomic number of the analyte. For small particle size samples, the characteristic radiation penetration depth increases. Thus, the probability of the particle size effect on the characteristic radiation decreases^20^. The presence of water in a slurry and its capability to absorb X-ray could affect the accuracy of the XRF measurements as well and can also cause the diffusion of primary radiation from excitation sources, decreasing of the intensity of the characteristic X-rays and an increase in the intensity of the X-rays scattered in the fluorescence spectrum^13^. Three certified reference materials with different water contents were analyzed with the XRF technique. The measurements show that the elements identified by the spectral line with the highest *Z* atomic number were more affected by the water content than the elements identified by the line with the lowest *Z*. Ti, Cr and Fe were not significantly influenced by water content, while the Sr was the most affected^21^. In some cases, the influence of water can be corrected by calculating the attenuation coefficients for each measured element^22^.

The work presented in this article is part of a project to develop an online XRF analyzer to control the quality of phosphate slurry directly on the production line. Particle size and water content are parameters that can adversely affect XRF measurements. Several studies have addressed the effect of these two parameters in the case of XRF analysis of other materials. However, the case of XRF analysis of phosphate slurry has not been reported in the literature, mainly because of the following parameters such as the specific chemical composition, mineralogical structure and other characteristics of the sample being analyzed. This study examines the effect of these two parameters on the XRF analysis of phosphate slurry. Seven samples of phosphate slurry with different particle sizes (106–425 µm) and sixteen samples with different water contents (from 30 to 60%) were analyzed. As a result of the data collected, new formulas will be proposed to correct the concentration based on particle size distribution and water content. Also, solutions are expected in the future to acquire these data in real-time to implement an XRF online analyzer system for phosphate slurry.

## Materials and methods

### Preparation of samples to investigate the effect of particle size distribution

The phosphate slurry samples were prepared in a laboratory based on dry phosphate ore provided by an international fertilizer producer. Six samples were prepared from the same batch of the ore weighing 2 kg. The sieving operation yielded to six samples of different particle sizes of 425 µm, 300 µm, 250 µm, 200 µm, 160 µm and 106 µm. These samples were dried in an oven for 12 h at 60 °C. The preparation of the slurry samples was done by adding 50% of water content to each sample for all the samples. Table 1 summarizes the characteristics of the six samples.

**Table 1:** The six samples prepared for studying the effect of particle size.

Sample	Brut_106	Brut_160	Brut_200	Brut_250	Brut_300	Brut_425
Particle size (µm)	106	160	200	250	300	425
Water content (%)	50	50	50	50	50	50

### Preparation of samples to investigate the effect of water content

Sixteen samples were used to study the effect of water content. They were prepared from four reference samples S1, S2, S3 and S4 provided by an international fertilizer producer. The four samples were ground to a fineness of 160 µm and dried for 12 h at a temperature of 60 °C. Based on each sample, four new slurry samples of different water content (30%, 40%, 50%, 60%) were prepared. In the industrial case, the phosphate slurry is often prepared with water content between 40 and 50% and transported between plant units through pipelines. In this study, the 30–60% water content range has been chosen to cover the real values used in industry. The preparation of the slurry samples was done by adding a well calculated amounts of water to the reference samples. Following this, the solutions were stirred for 5 min. Table 2 summarizes the 16 samples with water content.

**Table 2:** The 16 samples prepared for studying the effect of water content.

Basic samples ID	Prepared samples ID	Water content (%)	Particle size (µm)
Sample 1 (S1)	S1_60	60	160
	S1_50	50	160
	S1_40	40	160
	S1_30	30	160
Sample 2 (S2)	S2_60	60	160
	S2_50	50	160
	S2_40	40	160
	S2_30	30	160
Sample 3 (S3)	S3_60	60	160
	S3_50	50	160
	S3_40	40	160
	S3_30	30	160
Sample 4 (S4)	S4_60	60	160
	S4_50	50	160
	S4_40	40	160
	S4_30	30	160

### XRF setup

Each sample was divided into two sets for analysis by two different methods. The first set of samples was sent to external laboratories for analysis with conventional methods to determine the exact chemical composition of the samples and the concentrations of elements and chemical compounds. The second set of samples was analyzed using an XRF Epsilon 1 spectrometer (Malvern Panalytical, United Kingdom). The equipment measures the elemental concentrations, which are then converted to oxide concentrations. The results of XRF measurements will be compared with those of external laboratories and the relative error will be evaluated. The relative error $\Delta {w}_{i}(\%)$ expresses the ratio in percentage of the difference between the XRF measurement and the reference value measured by external laboratories. It can be expressed by the following equation.

1 $$\Delta w_{i} = \frac{{C_{ref} - C_{m} }}{{C_{ref} }}*100$$

where $C_{ref}$ the is validated reference concentration, $C_{m}$ the concentration measured by XRF, $x$ is the water content in percentage and *i* is the element measured by XRF.

**Figure 1:**
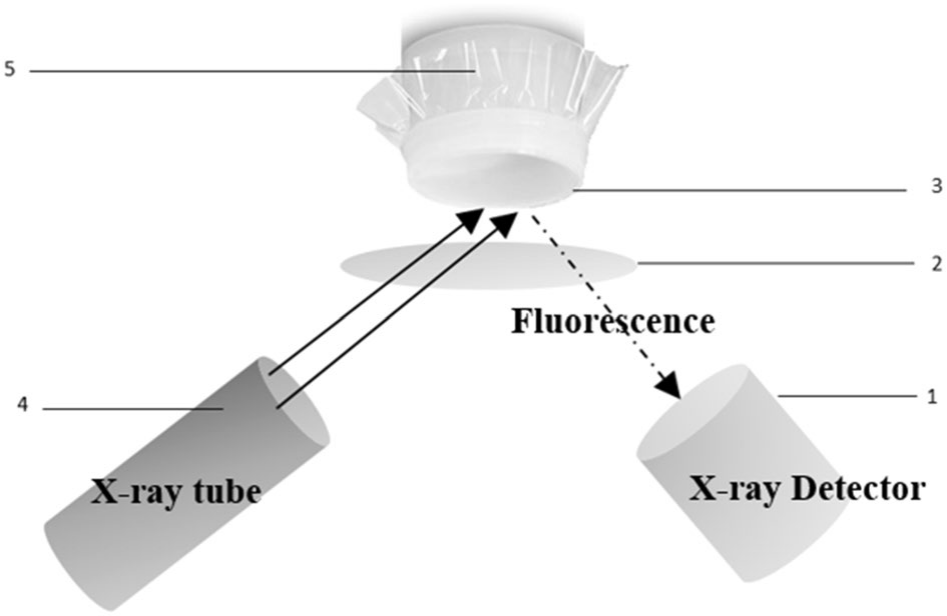
The main components of the XRF setup: (1) detector, (2) protection film, (3) 3.6 µm thick Mylar film, (4) X-ray tube, (5) cup sample holder.

The XRF Epsilon 1 equipment can analyze both solid and liquid samples. To analyze the slurry samples, plastic cups were used and the bottoms were covered with 3.6 µm thick Mylar polymer. Then, the slurry sample was poured into the cup and placed inside the XRF analyzer. Figure 1 shows the main components that make up the Epsilon 1 XRF analyzer. The X-ray tube has a power of 15 W with a 50 kV generator. The spot size on the sample is typically 10 × 14 mm. The detector has a high resolution of 135 eV. The matrix of phosphate slurry contains heavy and light elements at low and high concentrations. Thus, each sample was analyzed using three measurement parameter configurations, as shown in Table 3. Controlling measurement parameters such as excitation energy, electric current, filters and measurement time improves measurement accuracy. 10 kV energy is not sufficient to eject electrons from the K shell of elements with atomic number greater than 33. Therefore, the characteristic lines ${k}_{\alpha }$ of those elements do not appear on the spectrum, which decreases the probability of having a spectral overlap. Low current is often used for measuring metals, and high current for non-metals. The use of filters eliminates some characteristic lines and optimizes the signal-to-background ratio. The combination of excitation energy and different filters can make the instrument more sensitive to certain elements. The measurement time plays a significant role in the case of measuring light elements or elements with low concentrations, it improves the SNR ratio. More details about the configurations used in this study and the elements analysed by each configuration can be found in Appendix 2 of the supplementary material.

**Table 3:** The measurement parameters of the three configurations.

	Excitation energy (kV)	Current (µA)	Filter	Measurement time (S)	Elements measured
Config 1	50	100	Ag	100	As, Zn, U, Cu, Sr
Config 2	12	416	Al	120	K, Ca, Cr, Fe
Config 3	10	316	None	300	Al, Si, P, S, Cl

The effect of segregation (precipitation of the solid phase on the sample cup bottom) is considered to be relatively small for two reasons: the samples of the phosphate slurry that we prepared for this study have a high viscosity. Indeed, the mixture of dry phosphate rock with water remains homogeneous during the first 10 min. This is related to the characteristics of the phosphate rock such as the mineralogy, the matrix and the chemical composition. The second reason is that the XRF measurement is performed in a short time before the segregation. As shown in Table 3, each sample was analyzed using three measurement parameter configurations and sample homogenization is performed before each measurement. Configuration 3 is the longest and it takes 300 s to perform XRF measurements, precipitation is not expected to begin for quite some time after this duration.

**Figure 2:**
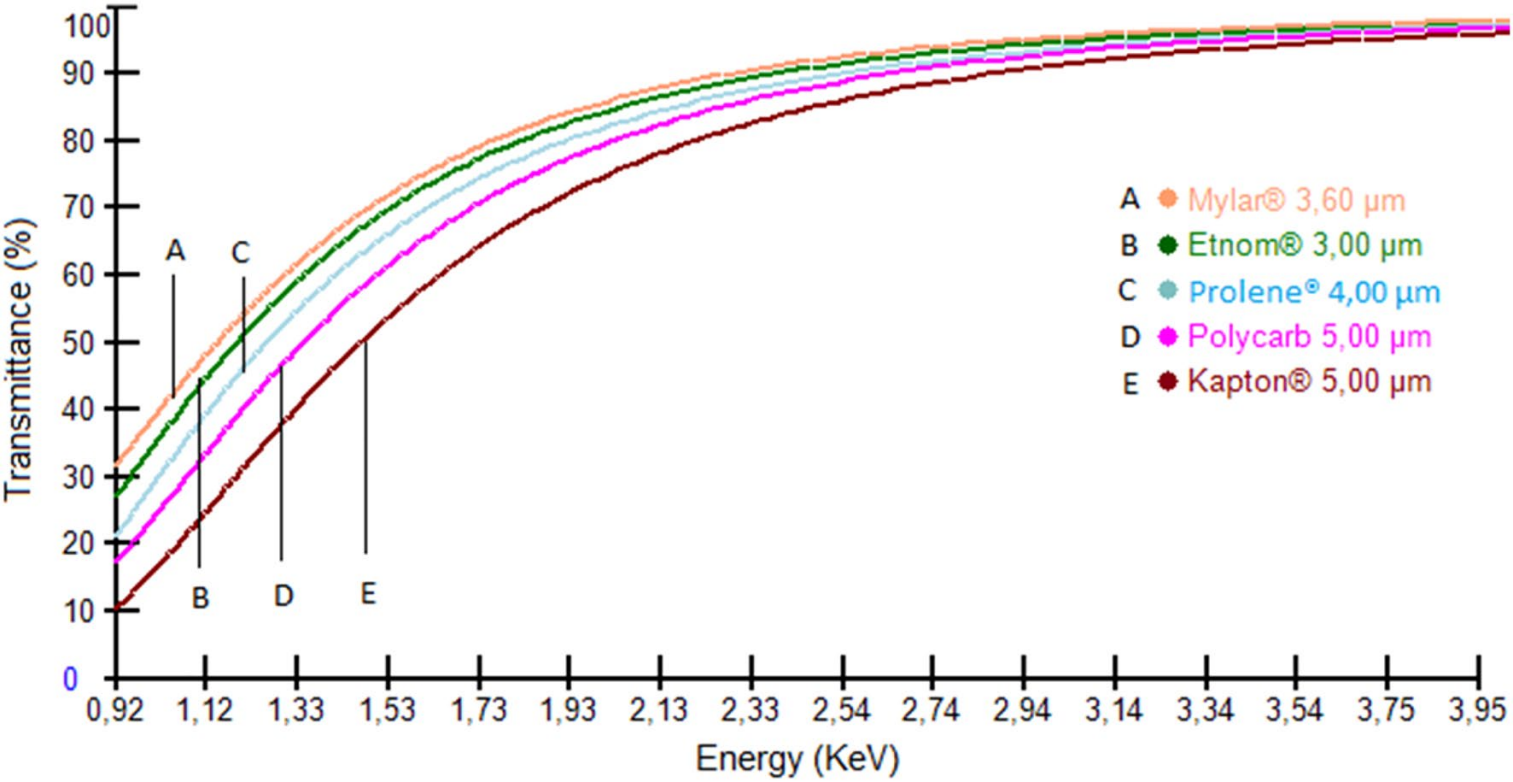
The plot of % transmittance vs wavelength for various thin films (taken from the Chemplex Thin-film Selection Guide, US patent 10,761,012).

Generally, the choice of the film is based on factors such as X-ray transmission, impurities, chemical resistance, and cost^23^. The analysis of some certified reference materials using the XRF technique showed that the energy absorption varies according to the material constituting the thin films^24^. The study confirmed that the transmittance of some light elements (Al, Mg, Si, K, S and Ca) has a high sensitivity to the thin film material. Generally, greater transmittance (minimal absorption) is observed for polypropylene (PP) than for Mylar. This difference is mainly explained by differences in density between the materials, Mylar being denser than PP. However, the film used in our study allows the transmittance of 65% K-line intensity of element Al, a transmittance of 75% K-line intensity of Si, and a transmittance greater than 80% of K-line intensity of other elements analyzed in this study, as shown in Fig. 2. Also, all light elements analyzed in this study are major elements and not trace elements. Thus, Mylar 3.6 µm film is suitable for this study.

## Results and discussion

### Equipment accuracy verification

To verify the XRF measurements taken with the Epsilon 1 spectrometer, four certified phosphate samples Mine1, Mine2, Mine3 and Mine4 were prepared and analyzed. Water was added to the powder samples to prepare slurry samples with a water content of 30% and a particle size of 106 µm. As a result of the measurements, the mean relative errors are as follows 5% for U, 12% for SO_3_, 15% for CaO, 18% for P_2_O_5_, 41% for SiO_2_, 23% for Fe_2_O_3_, 28% for Al_2_O_3_, 26% for Sr, 25 for Cu, 38% for Zn, 13% for As and 25% for Cl. The results are comparable to the certified laboratory analysis. Based on this first stage of measurements, this equipment was used for all XRF analyses. XRF analysis with different configurations listed in Table 3 allows the collection of spectra whose peaks of all elements are appropriate for accurate quantification. This section has been detailed in Appendix 2 of supplementary material.

### The effect of particle size distribution on XRF measurements

**Figure 3:**
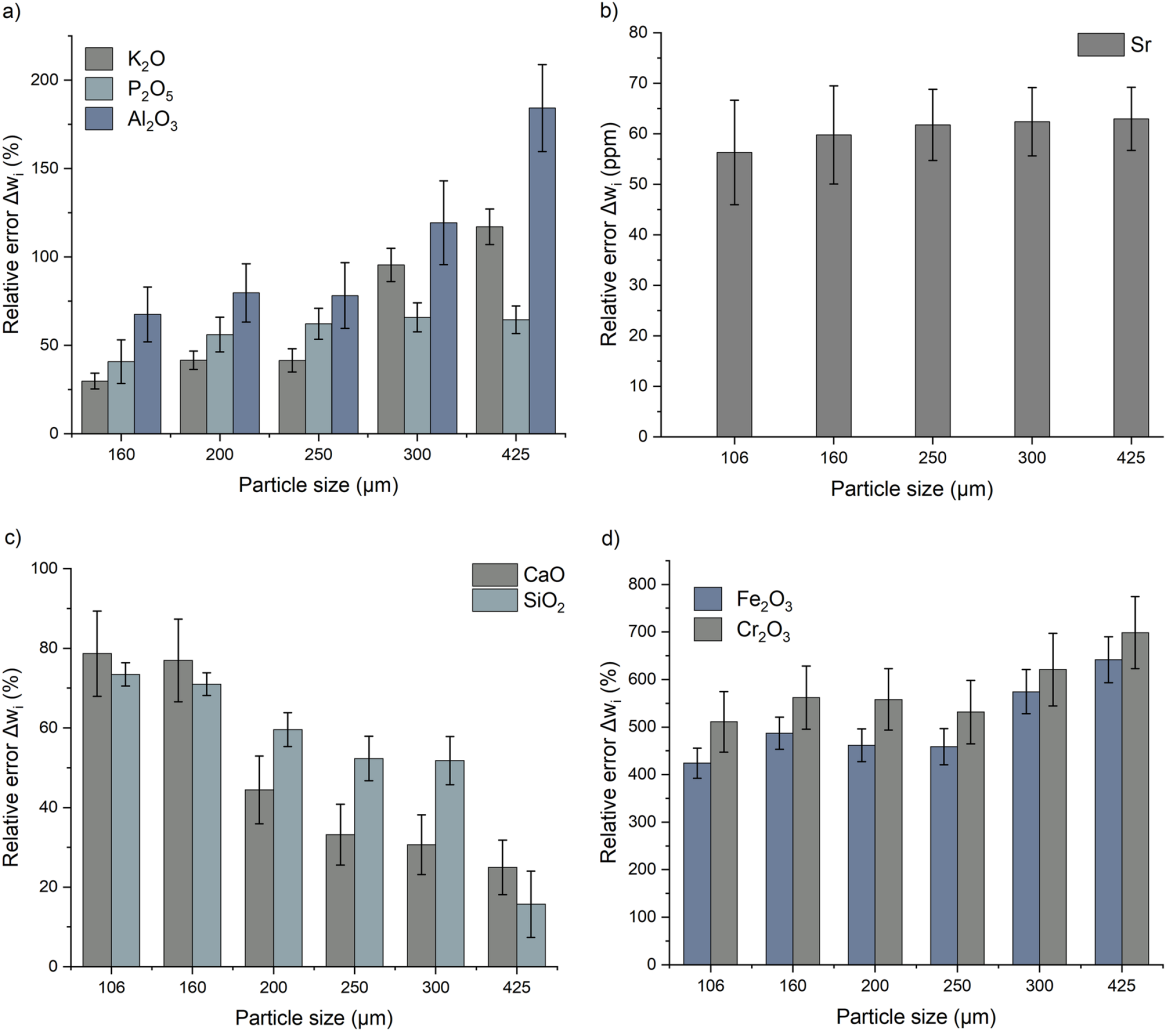
The relative error of XRF measurements as a function of particle size. (**a**) P_2_O_5_, Al_2_O_3_ and K_2_O, (**b**) Sr, (**c**) CaO and SiO_2_, (**d**) Fe_2_O_3_ and Cr_2_O_3_.

The six samples prepared for studying the effect of particle size were analyzed, a total of five spectra were collected for each sample. Table 4 shows the chemical analyzes performed by external laboratories and those using the XRF analyzer. It’s important to specify that compounds with lower concentrations (ppm) present large errors comparing the value measured using XRF and laboratory. Such behavior mainly occurs because of the special characteristics of these mineral slurry samples such as water that attenuate the X-ray intensities, the matrix, and the particle size. Analysis results of six samples show that the relative error varies depending on the particle size and the element being measured. For the P_2_O_5_ compound, the relative error increases when the particle size increases, as shown in Fig. 3a. With a particle size of 160 microns, the minimum error of XRF measurements is achieved, i.e. 3.45%. In the case of P_2_O_5_, the ratio between relative errors with 425 µm and 160 µm grain size was 1.53. The increase in P_2_O_5_ content as particle size increases could be interpreted as the larger particle size slurries segregating more rapidly. For the compounds Al_2_O_3_, K_2_O, Cr_2_O_3_, Fe_2_O_3_ and Sr, the results show that the relative error increases with the increase in particle size, as shown in Fig. 3a,b,d. The relative error increases by a factor of 1.11 for Sr, 1.36 for Cr_2_O_3_, 1.51 for Fe_2_O_3_, 4.01 for Al_2_O_3_ and 15.6 for K_2_O.While the measurements of CaO and SiO_2_ show a decrease in relative error with increasing particle size, as shown in Fig. 3c.

**Table 4:** Samples analysis of different grain sizes by external laboratories and by XRF.

Sample ID	Particle size µm	Analysis	SiO_2_%	Al_2_O_3_%	CaO %	K_2_O %	P_2_O_5_%	Sr ppm	Fe_2_O_3_%	Cr_2_O_3_%
Brut_106	106	Lab	19.0	0.68	13.9	0.24	8.15	387	0.22	0.01
		XRF	5.0	0.99	24.9	0.26	4.65	169	1.16	0.90
		$\Delta {w}_{i}(\%)$	73	46	79	8	43	56	424	511
Brut_160	160	Lab	19.2	0.64	14.4	0.22	8.35	412	0.20	0.01
		XRF	5.6	1.08	25.5	0.29	4.94	166	1.22	0.1
		$\Delta {w}_{i}(\%)$	71	68	77	30	41	60	487	562
Brut_200	200	Lab	13.0	0.60	17.6	0.18	10.5	510	0.20	0.01
		XRF	5.2	1.08	25.4	0.26	4.60	172	1.13	0.10
		$\Delta {w}_{i}(\%)$	60	80	44	42	56	66	462	558
Brut_250	250	Lab	9.9	0.53	19.6	0.15	11.8	570	0.18	0.01
		XRF	4.7	0.95	26.1	0.21	4.45	213	1.02	0.09
		$\Delta {w}_{i}(\%)$	52	78	33	42	62	63	459	531
Brut_300	300	Lab	9.1	0.42	20.0	0.10	12.4	590	0.15	0.01
		XRF	4.4	0.92	26.1	0.20	4.25	222	1.01	0.09
		$\Delta {w}_{i}(\%)$	52	119	31	95	66	62	574	621
Brut_425	425	Lab	6.6	0.40	21.9	0.09	13.3	640	0.14	0.01
		XRF	5.6	1.15	27.4	0.21	4.72	237	1.07	0.10
		$\Delta {w}_{i}(\%)$	16	184	25	117	64	63	642	698

This study's results can be expressed by the mineralogy of the phosphate rock that contains minerals with different characteristics. One of these characteristics is the size of the particles. For example, the sample Brut_106 with a particle size of 106 µm contains a higher amount of the minerals SiO_2_, Al_2_O_3_, K_2_O, Cr_2_O_3_ and Fe_2_O_3_ than the sample Brut_425 with a particle size of 425 µm. Table 4 shows that the contents of these chemical compounds decrease with increasing particle size. Alternatively, the concentrations of the compounds P_2_O_5_, Sr and CaO increase. The sample brut_425 is rich in tricalcium phosphate Ca_3_(PO_4_)_2_ but contains low levels of SiO_2_, Al_2_O_3_, K_2_O, Cr_2_O_3_ and Fe_2_O_3_. Some minerals are mostly found in the form of large particles and others in the form of small particles. Thus, different minerals do not have the same probabilities of being bombarded by the X-ray source beam. The claim that the P_2_O_5_ and CaO contents increased with particle size could also be interpreted as the larger particle size slurries segregating more rapidly. Indeed, the slurry samples will segregate on standing, or even during an XRF measurement and particles separate to the bottom with denser minerals more rapidly.

For most minerals, the relative error increases as the grain size increases. Indeed, a sample with a high particle size (e.g. brut_425) contains large particles of tricalcium phosphate Ca_3_(PO_4_)_2_ which cover a large part of the surface affected by the X-ray beam. In this case, the measured surface does not represent the exact composition of the measured sample. Alternatively, a sample with a small particle size (e.g., brut_106) contains minerals of small particles and the smallest particles of tricalcium phosphate. Thus, the minerals will have close probability of being bombarded by the x-ray source beam. However, this assumption does not explain the variation in relative error for the SiO_2_ compound. In contrast to other compounds, the relative error of XRF measurements decreases as the particle size increases. This could be explained by the fact that the XRF technique does not perform well for the light elements and the measurements are not precise as in the case of SiO_2_. This also justifies the random variation of the XRF measurements of the SiO_2_ content as a function of the particle size distribution of the samples.

**Figure 4:**
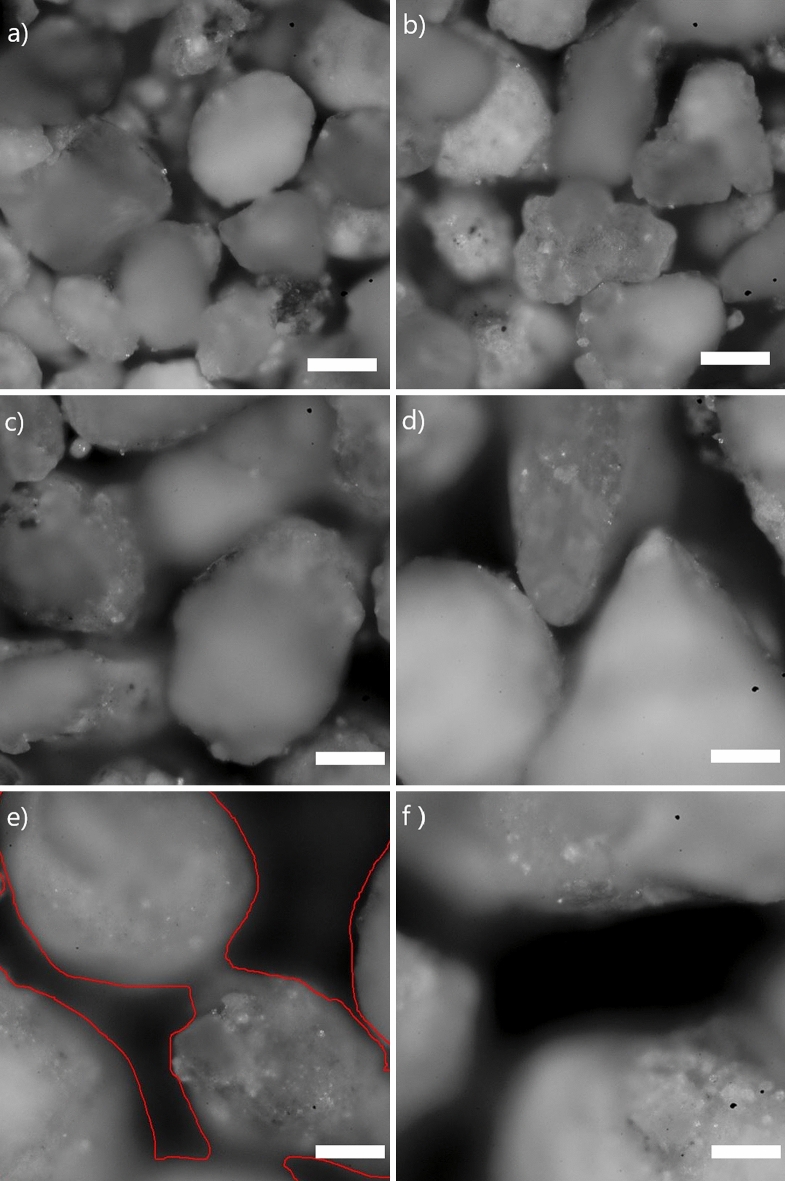
Microscopic images of the sample’s surface. (**a**) Particle size of 160 µm, (**b**) particle size of 200 µm, (**c**) particle size of 250 µm, (**d**) particle size of 300 µm, (**e**) particle size of 425 µm, (**f**) particle size of 500 µm. Scale bar in each figure represents 100 µm.

The other parameter that can express the increase in relative error as a function of grain size is the roughness of the measured surface. Indeed, the results are more precise when the measured surface is completely occupied by the particles or contains less empty area. The presence of an empty area on the measured surface makes it less representative of the measured sample. Usually, the empty area is filled by the water that absorbs the characteristic X-rays and affects the precision of the measurement. Visualization of the samples using a microscope shows that the empty area unoccupied by the particles increases as the particle size of the sample increases, as shown in Fig. 4.

**Figure 5:**
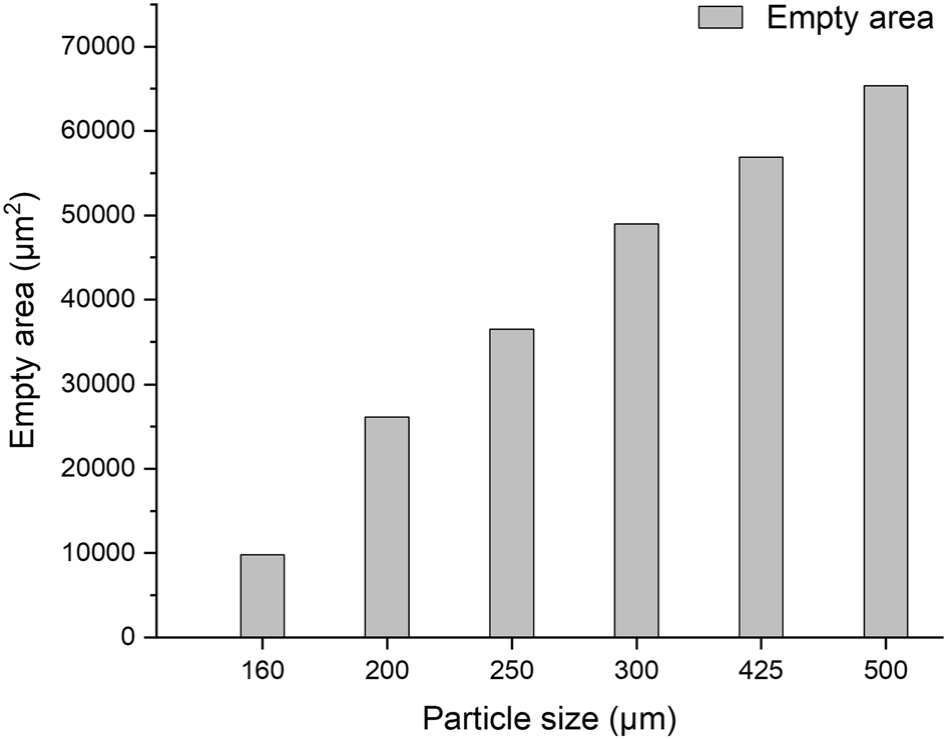
Evolution of the total empty area as a function of the particle size of the measured sample.

The unoccupied area was calculated using the microscope software. Figure 5 shows that this surface area increases as the particle size of the sample increases. This is a second parameter that may justify the increase in the relative error as the particle size of the sample increases.

The effect of particle size can be corrected using the Berry-Furuta-Rhodes equation of the characteristic X-ray intensity from a given element (), for particles of average diameter (cm) in a sample of arbitrary thickness (*n* + 1) layers of particles^25^.

2 $$I_{f} = \frac{{I_{0} G\omega \tau \rho_{f} W_{f} C\eta \left[ {1 - exp - \left( {\mu_{f}^{*} + \mu_{f}^{*^{\prime}} } \right)\overline{a}} \right]}}{{\left( {\mu_{f}^{*} + \mu_{f}^{*^{\prime}} } \right)}}\mathop \sum \limits_{k = 0}^{n} \left( {J,J^{\prime}} \right)^{k}$$

where $J = \left[ {1 - \eta + \eta C\left( {\exp - \left( {\mu_{f}^{*} \overline{a}} \right) + rexp - \left( {\mu_{n}^{*} \overline{a}} \right)} \right)} \right]$, $J^{\prime} = \left[ {1 - \eta + \eta C\left( {\exp - \left( {\mu_{f}^{*^{\prime}} \overline{a}} \right) + rexp - \left( {\mu_{n}^{*^{\prime}} \overline{a}} \right)} \right)} \right]$, *G* = geometrical constant, $I_{0}$ = flux of primary radiation at the sample surface (photons/s), $\omega$ = fluorescent yield for the X-ray transition excited, $\tau$ = photoelectric cross-section for that transition, at the source energy (cm^2^/g), $\rho_{f}$ = specific gravity of the fluorescent particles (g/cm^3^), $W_{f}$ = weight concentration of the fluorescent element in the fluorescent particles, *C* = volume concentration of fluorescent particles in the solid phase, $\mu_{f}^{*} ,\mu_{f}^{*^{\prime}}$ = linear attenuation coefficients for the primary and fluorescent radiation, respectively, in fluorescent particles (cm^−1^), $\mu_{n}^{*} ,\mu_{n}^{*^{\prime}}$ = the same, in non-fluorescent particles (cm^−1^), $\tau$ = total volume ratio of non-fluorescent to fluorescent particles, $\eta$ = ratio of total solid volume to the volume of the sample, i.e. the solids packing fraction ($\eta \le 1$).

### The effect of water content on XRF measurements

**Figure 6:**
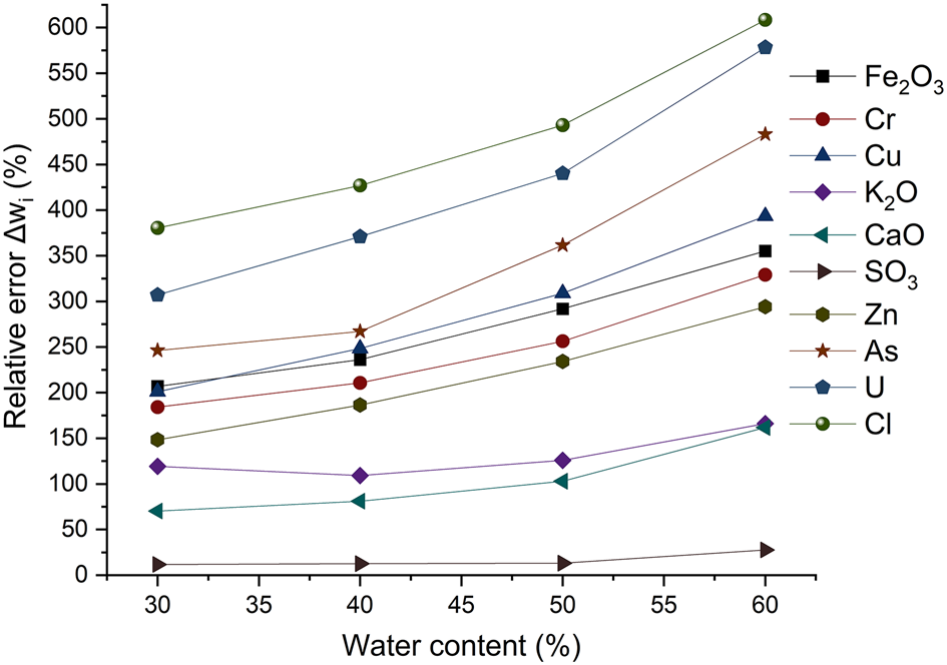
The relative error of XRF measurements as a function of water content, for all compounds contained in the phosphate slurry.

Appendix 1 represents the chemical analysis performed by the external laboratories and those using an XRF analyzer. For the compounds CaO, Cl, Fe_2_O_3_, K_2_O, As, Zn, U, Cr, SO_3_ and Cu, the results show that the relative error increases as the water content increases, as shown in Fig. 6. The ratio between the relative errors related to maximum and minimum grain sizes was 1.71 for Fe_2_O_3_, 1.78 for Cr, 1.95 for Cu, 1.39 for K_2_O, 2.30 for CaO, 2.35 for SO_3_, 1.98 for Zn, 1.96 for As, 1.88 for U and 1.59 for Cl. water content of 30% resulted in the minimum relative errors. The results can be expressed by the effect of water on X-rays. It causes a diffusion of primary radiation from excitation sources, decreasing the intensity of the characteristic X-rays and an increase in the intensity of the X-rays scattered in the fluorescence spectrum^13^. Therefore, the XRF measurement error increases for samples with high water content. However, this assumption does not explain the results of the P_2_O_5_ measurements. Indeed, the results do not show a clear correlation between the measurement error and the water content. It was found that a water content of 50% led to a minimum relative error of 14% as shown in Table 5.

**Table 5:** The relative error values for the XRF measurements of P_2_O_5_.

Water content (%)	Samples				Mean relative error
	S1	S2	S3	S4	
60	11	21	29	18	20
**50**	14	9	1	31	**14**
40	16	13	20	29	20
30	8	14	13	37	18

Significant values are in bold.

**Figure 7:**
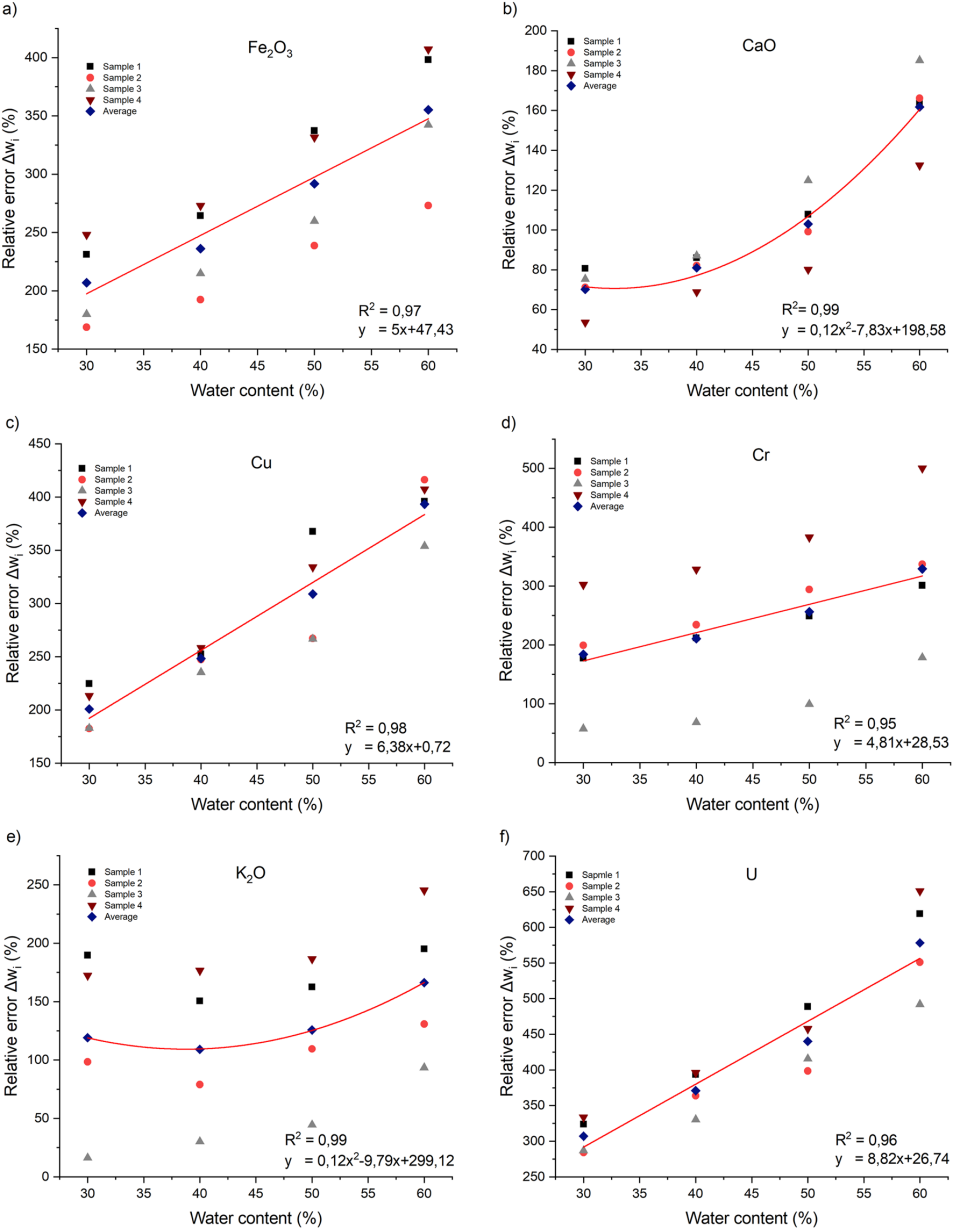

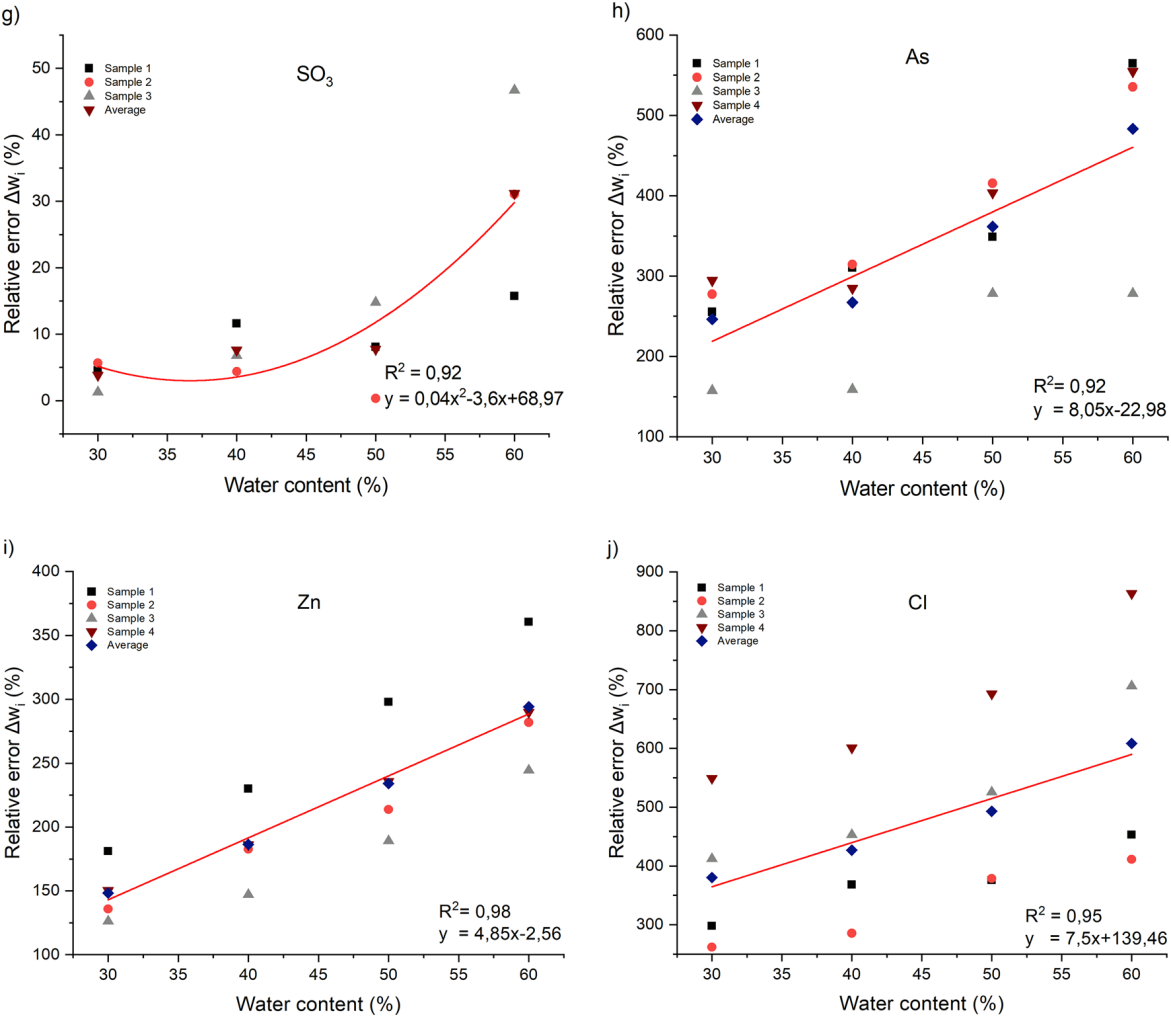
The relative error of XRF measurements as a function of water content. (**a**) Fe_2_O_3_, (**b**) CaO, (**c**) Cu, (**d**) Cr, (**e**) K_2_O, (**f**) U, (**g**) SO_3_, (**h**) As, (**i**) Zn, (**j**) Cl.

Figure 7 shows the linear and polynomial models that describe better the variation of the average relative error as a function of the water content for each element or compound. These models are important to correct the effect of water on the XRF measurements of phosphate slurry. It is important to specify that these models may vary depending on the equipment used and the measurement parameters. Considering that the water contents is between 30 and 60%. The equations of these models vary depending on the element or compound being measured.

3 $$\Delta w_{{Fe_{2} O_{3} }} \left( x \right) = 5x + 47,3$$

4 $$\Delta w_{Cr} \left( x \right) = 4,81x + 28,53$$

5 $$\Delta w_{Cu} \left( x \right) = 6,38x + 0,72$$

6 $$\Delta w_{{K_{2} O}} \left( x \right) = 0,12x^{2} - 9,79x + 299,12$$

7 $$\Delta w_{CaO} \left( x \right) = 0,12x^{2} - 7,83x + 198,58$$

8 $$\Delta w_{{SO_{3} }} \left( x \right) = 0,04x^{2} - 3,6x + 68,97$$

9 $$\Delta w_{Zn} \left( x \right) = 4,85x - 2,56$$

10 $$\Delta w_{As} \left( x \right) = 8,05x - 22,98$$

11 $$\Delta w_{U} \left( x \right) = 8,82x + 26,74$$

12 $$\Delta w_{Cl} \left( x \right) = 7,5x + 139,46$$

where $x$ is the water content in %, that is between 30 and 60%.

From to the definition of the relative error expressed by the formula (1), the measured concentration could be corrected by the following expression

13 $$C_{{cor_{\_i} }} \left( x \right) = \frac{Cm}{{1 - \frac{{\Delta w_{i} \left( x \right)}}{100}}}$$

where $C_{{cor_{\_i} }} \left( x \right)$ is the corrected concentration, $x$ is the water content in %, *i* is the element measured by XRF and $\Delta w_{i} \left( x \right)$ is one of the Eqs. (2)–(11) depending on the element being measured. It is important to specify that before correcting the effect of water content, the effect of grain size must first be corrected. The formula proposed in Eq. (2) can be used to calculate the corrected intensity taking into consideration the effect of particle size. Then, the elemental concentration will be calculated based on the corrected intensity. The new concentration will be corrected again using the formula proposed in Eq. (13) to consider the effect of water content.

## Conclusion

The effects of particle size and water content on XRF measurements of phosphate slurry were investigated in this study. The results showed that the relative error of XRF measurements increases as the particle size of the sample increases. This is for most compounds except SiO_2_ and CaO. The evolution of the error as a function of the grain size was quantified for the elements of interest contained in the phosphate slurry. The variation in the relative error as a function of the particle size could be explained by the mineralogy of the samples measured and the roughness of the measured surface. Also, this experimental study showed that the relative error of XRF measurements increases as the water content of the measured sample increases. This variation was expected, as the X-rays are absorbed by the water contained in the slurry. However, this assumption does not justify the results of the P_2_O_5_ measurements. Indeed, it was determined that 50% water content resulted in the least relative error. These results can be expressed by the mineralogy of the phosphate slurry, the size of the tricalcium phosphate Ca_3_(PO_4_)_2_ particles and the diameter of X-ray beams derived from the X-ray tube. For water content between 30 and 60%, the evolution of the relative error was represented by mathematical equations. Thus, new formulas were proposed to correct the concentration considering the water content.

## Supplementary Information

Supplementary Information.

## Data Availability

The datasets generated and/or analysed during the current study are available in the OneDrive Cloud repository, you can access all the data through this link.
