# Supplementary material for: The effect of particle size and water content on XRF measurements of phosphate slurry
Source: Sci Rep. 2022 Oct 24;12:17823. doi: 10.1038/s41598-022-21392-0 (PMC9592608; doi:10.1038/s41598-022-21392-0)
Supplement: Supplementary file 1 — Supplementary Information. [file 41598_2022_21392_MOESM1_ESM.docx]

**Supplementary material for**

**The effect of particle size and water content on XRF measurements of phosphate slurry**

**Ismail Ben Amar^1, 2^, Mourad Roudjane^3^, Hafid Griguer^2^, Amine Miled^1^, Younes Messaddeq^3^**

*^1^ Department of Electrical and Computer Engineering, Université Laval, Quebec City, Quebec, Canada*

*^2^ Mohammed VI Polytechnic University (UM6P), Ben Guerir, Morocco*

^3^ Center for Optics, Photonics, and Lasers, Université Laval, Quebec City, Quebec, Canada

**Tables of contents**

- Appendix 1: Chemical analyses obtained by external laboratories and using an XRF analyzer
- Appendix 2: Equipment accuracy verification

**Appendix 1**

**Chemical analyses obtained by external laboratories and using an XRF analyzer**

**Table 1** Chemical analyses obtained by external laboratories and using an XRF analyzer

| **Sample ID** | **Water %** | **Analysis** | **P_2_O_5_ %** | **CaO %** | **Cl  ppm** | **Fe_2_O_3_  %** | **K_2_O  %** | **SO_3_  %** | **As  ppm** | **Zn  ppm** | **U  ppm** | **Cr  ppm** | **Cu  ppm** |
| --- | --- | --- | --- | --- | --- | --- | --- | --- | --- | --- | --- | --- | --- |
| **S1_60** | 60% | Lab | 10.9 | 17.58 | 177 | 0.13 | 0.03 | 0.59 | 3.3 | 77.4 | 52.2 | 81.5 | 10.4 |
|  |  | XRF | 12.1 | 46.33 | 977 | 0.66 | 0.1 | 0.68 | 21.8 | 356.5 | 375.3 | 326.8 | 51.5 |
|  |  | $\Delta w_{i}\boldsymbol{(\%)}$ | 11 | 164 | 453 | 408 | 233 | 15 | 565 | 361 | 619 | 301 | 396 |
| **S1_50** | 50% | Lab | 13.63 | 21.97 | 221 | 0.16 | 0.04 | 0.74 | 4.1 | 96.8 | 65.3 | 101.8 | 13.0 |
|  |  | XRF | 11.77 | 45.66 | 1050 | 0.72 | 0.11 | 0.68 | 18.4 | 385.1 | 384.2 | 355.5 | 60.7 |
|  |  | $\Delta w_{i}\boldsymbol{(\%)}$ | 14 | 108 | 376 | 350 | 175 | 8 | 349 | 298 | 489 | 249 | 368 |
| **S1_40** | 40% | Lab | 16.36 | 26.37 | 265 | 0.19 | 0.05 | 0.89 | 4.9 | 116.1 | 78.3 | 122.2 | 15.6 |
|  |  | XRF | 13.76 | 49.04 | 1240 | 0.72 | 0.13 | 0.78 | 20.2 | 383.1 | 386.5 | 380.9 | 54.9 |
|  |  | $\Delta w_{i}\boldsymbol{(\%)}$ | 16 | 86 | 368 | 279 | 160 | 12 | 311 | 230 | 394 | 212 | 253 |
| **S1_30** | 30% | Lab | 19.09 | 30.76 | 309 | 0.23 | 0.06 | 1.03 | 5.7 | 135.5 | 91.4 | 142.6 | 18.2 |
|  |  | XRF | 17.65 | 55.57 | 1230 | 0.77 | 0.17 | 0.99 | 20.4 | 380.7 | 387.1 | 395.3 | 59.0 |
|  |  | $\Delta w_{i}\boldsymbol{(\%)}$ | 8 | 81 | 298 | 235 | 183 | 4 | 255 | 181 | 324 | 177 | 225 |
| **S2_60** | 60% | Lab | 12.33 | 20.26 | 149 | 0.11 | 0.03 | 0.62 | 4.4 | 96.4 | 54.9 | 69.5 | 14.7 |
|  |  | XRF | 14.92 | 53.92 | 763 | 0.43 | 0.08 | 0.81 | 27.7 | 368.3 | 357.7 | 303.6 | 75.9 |
|  |  | $\Delta w_{i}\boldsymbol{(\%)}$ | 21 | 166 | 411 | 291 | 167 | 31 | 535 | 282 | 551 | 337 | 416 |
| **S2_50** | 50% | Lab | 15.41 | 25.32 | 186 | 0.14 | 0.04 | 0.77 | 5.5 | 120.6 | 68.7 | 86.8 | 18.4 |
|  |  | XRF | 14.06 | 50.43 | 893 | 0.49 | 0.09 | 0.78 | 28.1 | 378.1 | 342.3 | 342.3 | 67.5 |
|  |  | $\Delta w_{i}\boldsymbol{(\%)}$ | 9 | 99 | 379 | 250 | 125 | 1 | 416 | 214 | 399 | 294 | 267 |
| **S2_40** | 40% | Lab | 18.5 | 30.39 | 224 | 0.17 | 0.05 | 0.93 | 6.5 | 144.7 | 82.4 | 104.2 | 22.1 |
|  |  | XRF | 16.09 | 55.35 | 863 | 0.51 | 0.09 | 0.89 | 27.1 | 408.8 | 382.1 | 348.4 | 76.6 |
|  |  | $\Delta w_{i}\boldsymbol{(\%)}$ | 13 | 82 | 286 | 200 | 80 | 4 | 314 | 183 | 364 | 234 | 247 |
| **S2_30** | 30% | Lab | 21.58 | 35.45 | 261 | 0.2 | 0.06 | 1.09 | 7.6 | 168.8 | 96.1 | 121.6 | 25.7 |
|  |  | XRF | 18.57 | 60.7 | 945 | 0.55 | 0.12 | 1.02 | 28.8 | 398.1 | 369.1 | 363.8 | 72.7 |
|  |  | $\Delta w_{i}\boldsymbol{(\%)}$ | 14 | 71 | 262 | 175 | 100 | 6 | 277 | 136 | 284 | 199 | 183 |
| **S3_60** | 60% | Lab | 11.73 | 19.44 | 103 | 0.05 | 0.02 | 0.61 | 4.0 | 91.4 | 60.4 | 47.8 | 13.6 |
|  |  | XRF | 15.18 | 55.44 | 832 | 0.22 | 0.04 | 0.9 | 15.2 | 314.7 | 357.8 | 133.1 | 61.8 |
|  |  | $\Delta w_{i}\boldsymbol{(\%)}$ | 29 | 185 | 706 | 340 | 100 | 48 | 279 | 244 | 492 | 178 | 354 |
| **S3_50** | 50% | Lab | 14.67 | 24.3 | 129 | 0.06 | 0.03 | 0.768 | 5.0 | 114.2 | 75.6 | 59.8 | 17.0 |
|  |  | XRF | 14.5 | 54.64 | 807 | 0.23 | 0.04 | 0.88 | 19.0 | 330.2 | 389.6 | 118.9 | 62.4 |
|  |  | $\Delta w_{i}\boldsymbol{(\%)}$ | 1 | 125 | 525 | 283 | 33 | 15 | 278 | 189 | 416 | 99 | 267 |
| **S3_40** | 40% | Lab | 17.6 | 29.17 | 155 | 0.07 | 0.03 | 0.92 | 6.0 | 137.1 | 90.7 | 71.7 | 20.4 |
|  |  | XRF | 14.06 | 54.57 | 857 | 0.24 | 0.05 | 0.85 | 15.6 | 338.5 | 390.2 | 120.6 | 68.5 |
|  |  | $\Delta w_{i}\boldsymbol{(\%)}$ | 20 | 87 | 453 | 243 | 67 | 8 | 159 | 147 | 330 | 68 | 235 |
| **S3_30** | 30% | Lab | 20.54 | 34.03 | 181 | 0.08 | 0.04 | 1.07 | 7.0 | 159.9 | 105.8 | 83.7 | 23.8 |
|  |  | XRF | 17.96 | 59.67 | 926 | 0.25 | 0.05 | 1.08 | 18.1 | 361.6 | 409.1 | 131.7 | 67.4 |
|  |  | $\Delta w_{i}\boldsymbol{(\%)}$ | 13 | 75 | 412 | 213 | 25 | 1 | 157 | 126 | 287 | 57 | 183 |
| **S4_60** | 60% | Lab | 10.82 | 18.94 | 80 | 0.17 | 0.04 | 0.51 | 4.8 | 135.2 | 51.8 | 101.8 | 14.9 |
|  |  | XRF | 8.9 | 44.01 | 769 | 0.88 | 0.16 | 0.43 | 31.2 | 527.1 | 389.2 | 611.1 | 75.8 |
|  |  | $\Delta w_{i}\boldsymbol{(\%)}$ | 18 | 132 | 863 | 418 | 300 | 16 | 555 | 290 | 651 | 500 | 407 |
| **S4_50** | 50% | Lab | 13.53 | 23.67 | 100 | 0.21 | 0.05 | 0.64 | 6.0 | 169.0 | 64.8 | 127.3 | 18.7 |
|  |  | XRF | 9.33 | 42.64 | 790 | 0.94 | 0.16 | 0.45 | 30.0 | 567.3 | 361.3 | 614.5 | 81.1 |
|  |  | $\Delta w_{i}\boldsymbol{(\%)}$ | 31 | 80 | 692 | 348 | 220 | 30 | 404 | 236 | 458 | 383 | 334 |
| **S4_40** | 40% | Lab | 16.23 | 28.41 | 120 | 0.26 | 0.07 | 0.77 | 7.1 | 202.8 | 77.8 | 152.8 | 22.4 |
|  |  | XRF | 11.51 | 47.94 | 839 | 0.97 | 0.19 | 0.56 | 27.5 | 580.3 | 385.7 | 654.1 | 80.3 |
|  |  | $\Delta w_{i}\boldsymbol{(\%)}$ | 29 | 69 | 601 | 273 | 171 | 27 | 285 | 186 | 396 | 328 | 258 |
| **S4_30** | 30% | Lab | 18.94 | 33.14 | 140 | 0.3 | 0.08 | 0.9 | 8.3 | 236.7 | 90.7 | 178.2 | 26.1 |
|  |  | XRF | 11.96 | 50.89 | 906 | 1.06 | 0.22 | 0.58 | 32.9 | 592.4 | 393.2 | 716.7 | 81.9 |
|  |  | $\Delta w_{i}\boldsymbol{(\%)}$ | 37 | 54 | 549 | 253 | 175 | 36 | 294 | 150 | 333 | 302 | 213 |

**Appendix 2**

**Equipment accuracy verification**

To verify the XRF measurements taken with the Epsilon 1 spectrometer, four certified phosphate samples Mine1, Mine2, Mine3 and Mine4 were prepared and analyzed. Water was added to the powder samples to prepare slurry samples with a water content of 30% and a particle size of 106 µm. As a result of the measurements, the mean relative error is as follows 5% for U, 12% for SO_3_, 15% for CaO, 18% for P_2_O_5_, 41% for SiO_2_, 23% for Fe_2_O_3_, 28% for Al_2_O_3_, 26% for Sr, 25 for Cu, 38% for Zn, 13% for As and 25% for Cl. The results are comparable to the certified laboratory analysis. Based on this first stage of measurements, this equipment was used for all XRF analyses.

Table 2 shows the elemental concentrations obtained directly by the analyzer Epsilon 1. Correction factors were applied to correct the overestimated elemental concentrations. Since the equipment was often used last few years to analyze the phosphate slurry, The value of correction factors is already known. Table 2 represents the values of the correction factors and the elemental concentrations after the correction.

**Table 2** Elemental concentrations before and after correction

|  | **Sample ID** | **Al %** | **Si %** | **P %** | **S %** | **Cl ppm** | **K %** | **Ca %** | **Cr ppm** | **Fe %** | **Cu ppm** | **Zn ppm** | **As ppm** | **Sr ppm** | **U ppm** |
| --- | --- | --- | --- | --- | --- | --- | --- | --- | --- | --- | --- | --- | --- | --- | --- |
| Elemental concentration | **Mine 1** | 0.88 | 2.67 | 7.70 | 0.40 | 1230 | 0.15 | 39.72 | 395 | 0.60 | 59 | 381 | 20 | 2240 | 387 |
|  | **Mine 2** | 0.68 | 1.08 | 8.10 | 0.41 | 945 | 0.10 | 43.38 | 364 | 0.43 | 73 | 398 | 29 | 1630 | 369 |
|  | **Mine 3** | 0.39 | 0.61 | 7.84 | 0.44 | 926 | 0.04 | 42.65 | 132 | 0.20 | 67 | 362 | 18 | 2390 | 409 |
|  | **Mine 4** | 1.02 | 2.72 | 5.22 | 0.24 | 906 | 0.19 | 36.37 | 717 | 0.83 | 82 | 592 | 33 | 1720 | 393 |
| Correction factors | | **0,25** | **1** | **1** | **1** | **0.25** | **0.25** | **0.5** | **0.25** | **0.25** | **0.25** | **0.25** | **0.25** | **0.25** | **0.25** |
| Corrected elemental concentration | **Mine 1** | 0.22 | 2.67 | 7.70 | 0.40 | 308 | 0.04 | 19.86 | 99 | 0.15 | 15 | 95 | 5 | 560 | 97 |
|  | **Mine 2** | 0.17 | 1.08 | 8.10 | 0.41 | 236 | 0.03 | 21.69 | 91 | 0.11 | 18 | 100 | 7 | 408 | 92 |
|  | **Mine 3** | 0.10 | 0.61 | 7.84 | 0.44 | 232 | 0.01 | 21.32 | 33 | 0.05 | 17 | 90 | 5 | 598 | 102 |
|  | **Mine 4** | 0.25 | 2.72 | 5.22 | 0.24 | 227 | 0.05 | 18.19 | 179 | 0.21 | 20 | 148 | 8 | 430 | 98 |

The spectra of a certified sample analyzed with the three configurations are shown in Fig. 1-6. The elemental concentrations were converted into oxide concentrations by multiplying them by conversion coefficients shown in Table 3. The concentrations of oxides are shown in Table 4. The light elements in the range 1.0 to 3.0 KeV are Al, Si, P, S and Cl. Measuring the sample with configuration 3 (10 kV, 316 µA, none filter, 100 s) allows to clearly see the $k_{\alpha}$ lines of these elements as shown in Fig. 1. The intensity of the $k_{\alpha}$line of Aluminum in Fig. 1 is weak but the peak is still visible. Also, the analyzes of the four certified samples show that it was possible to quantify Al with a relative error of 28% as shown in Table 5.

Configuration 2 (12 kV, 416 µA, Al filter, 120 s) was used to analyse the elements K, Ca, Cr, Fe, the spectra is shown in the Fig. 2, the the $k_{\alpha}$ lines of Cr and K are highlighted with a zoom in in Fig.3.


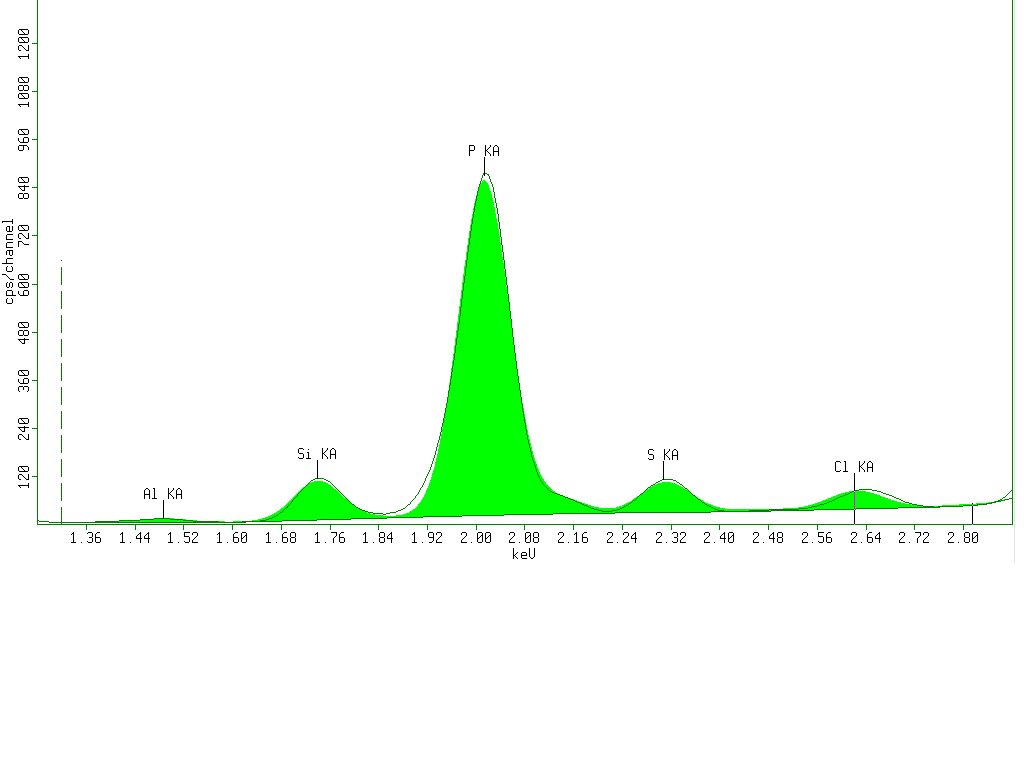


**Fig. 1** Spectra of a certified sample, range 1.0 to 3 KeV, measurement taken with config 3 (10 kV, 316 µA, none filter, 100 s)


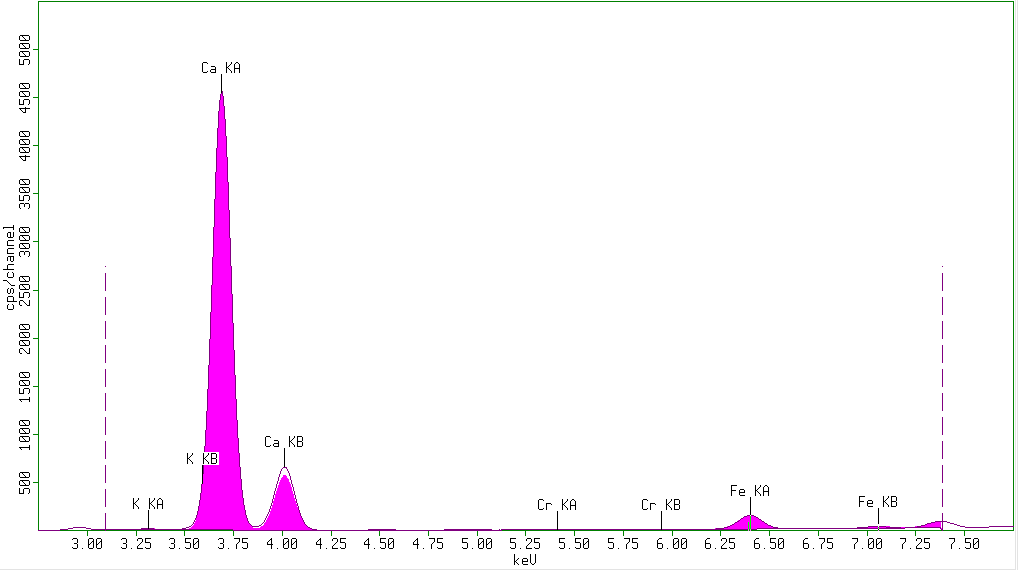


**Fig. 2** Spectra of a certified sample measured with config 2 (12 kV, 416 µA, Al filter, 120 s), range 0.0 to 5000 cps/ channel


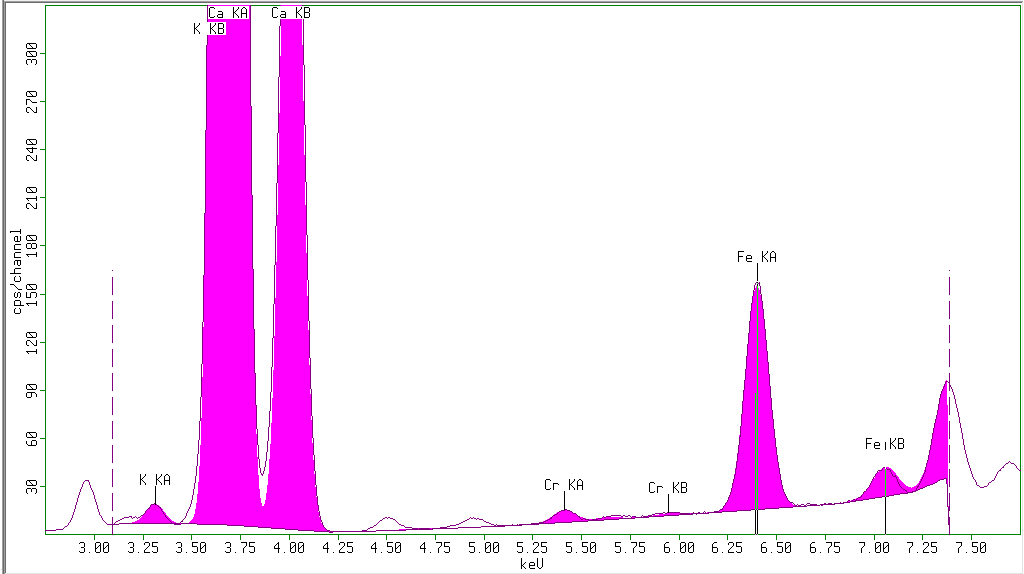


**Fig. 3** Spectra of a certified sample measured with config 2 (12 kV, 416 µA, Al filter, 120 s), range 0.0 to 350 cps/ channel

Configuration 1 (50 kV, 100 µA, Ag filter, 100 s) was used to analyse the elements As, Zn, U, Cu and Sr, the spectra is shown in the Fig. 4, the the $k_{\alpha}$ lines of Cu, Zn, As and U are highlighted with a zoom in in Fig.5.


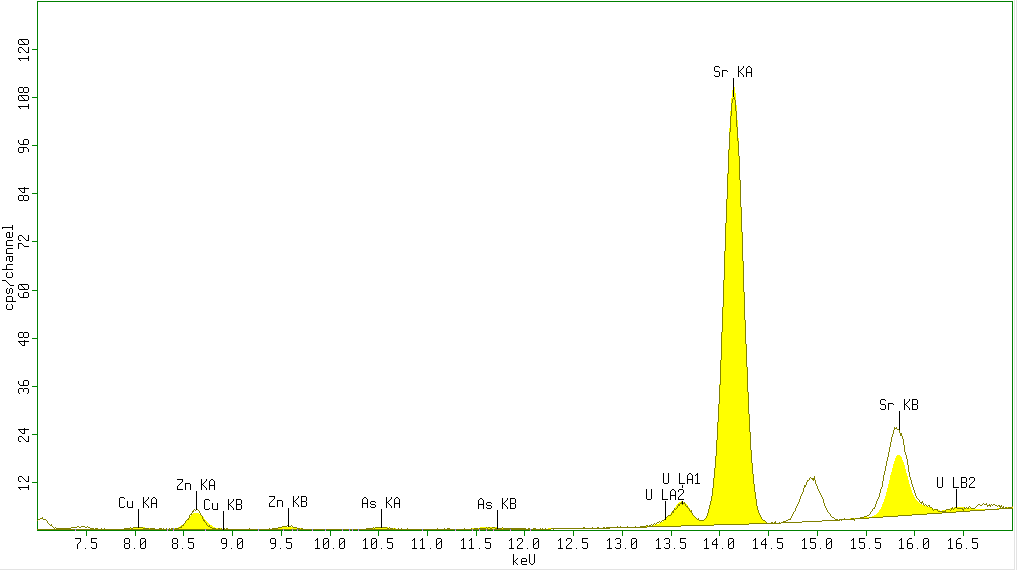


**Fig. 4** Spectra of a certified sample measured with config 1 (50 kV, 100 µA, Ag filter, 100 s), range 0.0 to 120 cps/ channel


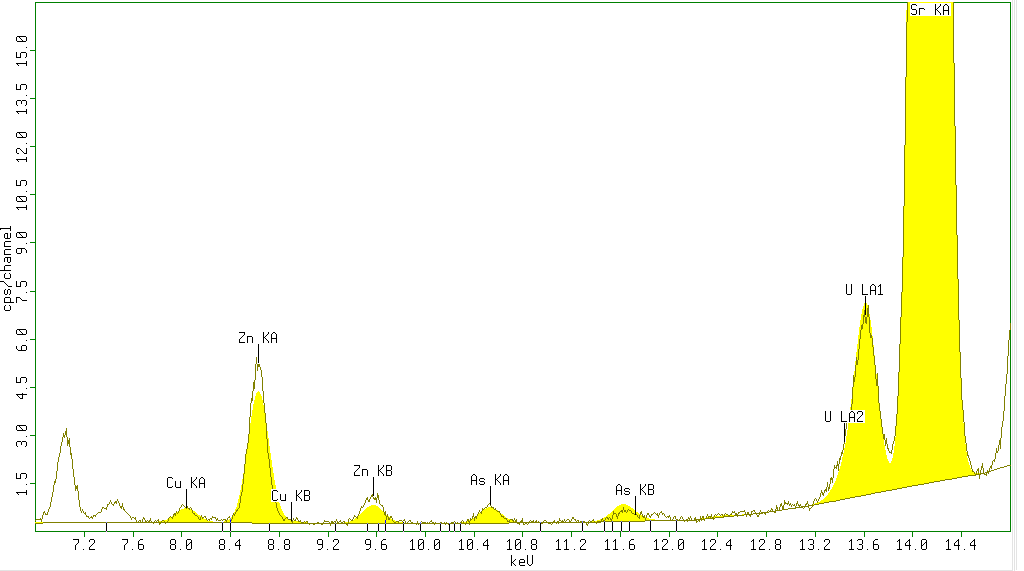


**Fig. 5** Spectra of a certified sample measured with config 2 (12 kV, 416 µA, Al filter, 120 s), range 0.0 to 15 cps/ channel


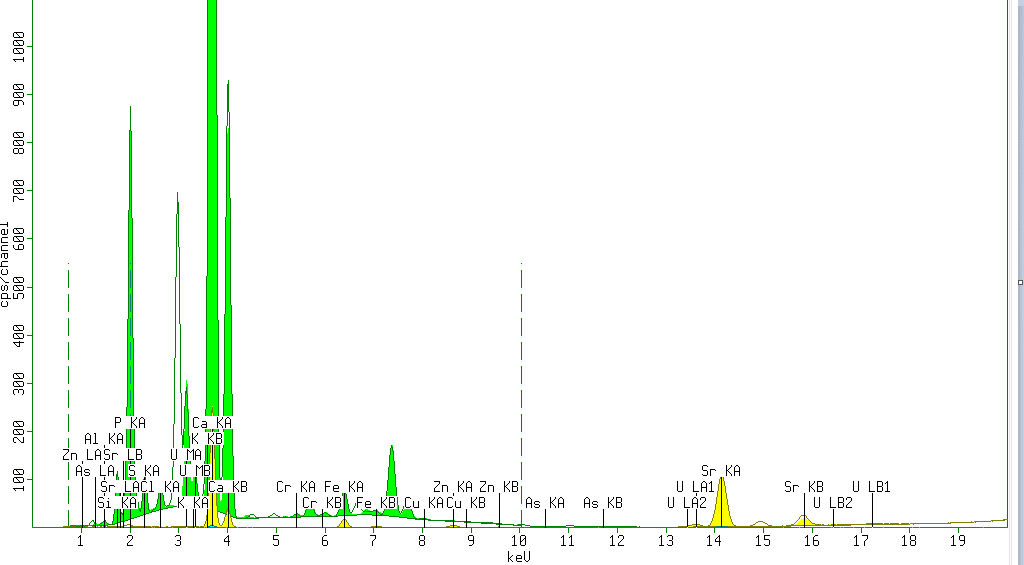


**Fig. 6** Spectra of a certified sample, range 1.0 to 18 KeV, measurement taken with three configurations

**Table 3** Element to oxide conversion coefficients

| **Conversion coefficients** | |
| --- | --- |
| Al to Al_2_O_3_ | 1.8895 |
| Si to SiO_2_ | 2.1392 |
| P to P_2_O_5_ | 2.2916 |
| S to SO_3_ | 2.4972 |
| K to K_2_O | 1.2046 |
| Ca to CaO | 1.3992 |
| Cr to Cr_2_O_3_ | 1.462 |
| Fe to Fe_2_O_3_ | 1.2865 |
| Zn to ZnO | 1.2447 |

**Table 4** Concentrations of oxides

| **Sample ID** | **Solid content** | **Particle size** | **P_2_O_5_ %** | **CaO %** | **SiO_2_ %** | **Cl ppm** | **Al_2_O_3_ %** | **Fe_2_O_3_ %** | **K_2_O %** | **SO_3_ %** | **As ppm** | **Zn ppm** | **U ppm** | **Cr ppm** | **Cu ppm** | **Sr ppm** |
| --- | --- | --- | --- | --- | --- | --- | --- | --- | --- | --- | --- | --- | --- | --- | --- | --- |
| **Mine1** | 55% | 106 µm | 17.65 | 27.79 | 5.70 | 308 | 0.42 | 0.19 | 0.04 | 0.99 | 5 | 95 | 97 | 99 | 15 | 560 |
| **Mine2** | 55% | 106 µm | 18.57 | 30.35 | 2.32 | 236 | 0.32 | 0.14 | 0.03 | 1.03 | 7 | 100 | 92 | 91 | 18 | 408 |
| **Mine3** | 55% | 106 µm | 17.97 | 29.84 | 1.31 | 232 | 0.19 | 0.06 | 0.01 | 1.09 | 5 | 90 | 102 | 33 | 17 | 598 |
| **Mine4** | 55% | 106 µm | 11.96 | 25.45 | 5.82 | 227 | 0.48 | 0.27 | 0.06 | 0.59 | 8 | 148 | 98 | 179 | 20 | 430 |

Table 5 shows the reference values of certified samples and XRF analysis performed using Epsilon 1 equipment.

**Table 5** reference values of certified samples and XRF analysis

| **Sample ID** | **Analysis** | **P_2_O_5_ %** | **CaO %** | **SiO_2_ %** | **Cl ppm** | **Al_2_O_3_ %** | **Fe_2_O_3_ %** | **K_2_O %** | **SO_3_ %** | **As ppm** | **Zn ppm** | **U ppm** | **Cr ppm** | **Cu ppm** | **Sr ppm** |
| --- | --- | --- | --- | --- | --- | --- | --- | --- | --- | --- | --- | --- | --- | --- | --- |
| **Mine1** | Ref | 19.09 | 30.76 | 9.25 | 309 | 0.52 | 0.23 | 0.06 | 1.04 | 5.7 | 135 | 91.4 | 143 | 18.2 | 713 |
|  | XRF | 17.65 | 27.79 | 5.70 | 308 | 0.42 | 0.19 | 0.04 | 0.99 | 5.1 | 95 | 96.8 | 99 | 14.8 | 560 |
|  | Error | 1.44 | 2.97 | 3.55 | 2 | 0.10 | 0.04 | 0.02 | 0.05 | 0.6 | 40 | 5.4 | 44 | 3.4 | 153 |
|  | $\Delta_{\omega_{i}}(\%)$ | 8 | 10 | 38 | 1 | 19 | 17 | 28 | 5 | 11 | 30 | 6 | 31 | 19 | 21 |
| **Mine2** | Ref | 21.59 | 35.45 | 3.01 | 261 | 0.50 | 0.21 | 0.06 | 1.09 | 7.6 | 169 | 96.1 | 122 | 25.7 | 553 |
|  | XRF | 18.57 | 30.35 | 2.32 | 236 | 0.32 | 0.14 | 0.03 | 1.03 | 7.2 | 100 | 92.3 | 91 | 18.2 | 408 |
|  | Error | 3.01 | 5.10 | 0.69 | 25 | 0.18 | 0.07 | 0.03 | 0.06 | 0.4 | 69 | 3.9 | 31 | 7.6 | 146 |
|  | $\Delta_{\omega_{i}}(\%)$ | 14 | 14 | 23 | 9 | 36 | 33 | 50 | 6 | 6 | 41 | 4 | 25 | 29 | 26 |
| **Mine3** | Ref | 20.54 | 34.03 | 4.77 | 181 | 0.23 | 0.09 | 0.05 | 1.08 | 7.0 | 160 | 105.8 | 84 | 23.8 | 825 |
|  | XRF | 17.97 | 29.84 | 1.31 | 232 | 0.19 | 0.06 | 0.01 | 1.09 | 4.5 | 90 | 102.3 | 33 | 16.9 | 598 |
|  | Error | 2.58 | 4.19 | 3.46 | 51 | 0.04 | 0.03 | 0.03 | 0.01 | 2.5 | 69 | 3.5 | 51 | 7.0 | 228 |
|  | $\Delta_{\omega_{i}}(\%)$ | 13 | 12 | 72 | 28 | 19 | 30 | 71 | 1 | 36 | 43 | 3 | 61 | 29 | 28 |
| **Mine4** | Ref | 18.94 | 33.14 | 4.46 | 140 | 0.76 | 0.31 | 0.08 | 0.91 | 8.3 | 237 | 90.7 | 178 | 26.1 | 612 |
|  | XRF | 11.96 | 25.45 | 5.82 | 227 | 0.48 | 0.27 | 0.06 | 0.59 | 8.2 | 148 | 98.3 | 179 | 20.5 | 430 |
|  | Error | 6.98 | 7.69 | 1.36 | 87 | 0.28 | 0.04 | 0.03 | 0.32 | 0.1 | 89 | 7.6 | 1 | 5.7 | 182 |
|  | $\Delta_{\omega_{i}}(\%)$ | 37 | 23 | 30 | 62 | 36 | 13 | 32 | 35 | 1 | 37 | 8 | 1 | 22 | 30 |
| Mean error | | 3,50 | 4.99 | 2.27 | 41 | 0.15 | 0.04 | 0.03 | 0.11 | 1 | 67 | 5 | 32 | 6 | 177 |
| **Mean relative error** | | **18** | **15** | **41** | **25** | **28** | **23** | **45** | **12** | **13** | **38** | **5** | **29** | **25** | **26** |
